# Draft genome sequence of *Streptomyces hyaluromycini* MB-PO13^T^, a hyaluromycin producer

**DOI:** 10.1186/s40793-017-0286-7

**Published:** 2018-01-11

**Authors:** Enjuro Harunari, Hisayuki Komaki, Natsuko Ichikawa, Akira Hosoyama, Akane Kimura, Moriyuki Hamada, Yasuhiro Igarashi

**Affiliations:** 1 Biotechnology Research Center and Department of Biotechnology, Toyama Prefectural University, Toyama, Japan; 2 Biological Resource Center, National Institute of Technology and Evaluation (NBRC), Chiba, Japan; 3 NBRC, Tokyo, Japan

**Keywords:** Biosynthesis, C_5_N, Polyketide synthase, Rubromycin, *Streptomyces*

## Abstract

*Streptomyces hyaluromycini* MB-PO13^T^ (=NBRC 110483^T^ = DSM 100105^T^) is type strain of the species, which produces a hyaluronidase inhibitor, hyaluromycin. Here, we report the draft genome sequence of this strain together with features of the organism and generation, annotation and analysis of the genome sequence. The 11.5 Mb genome of *Streptomyces hyaluromycini* MB-PO13^T^ encoded 10,098 putative ORFs, of which 5317 were assigned with COG categories. The genome harbored at least six type I PKS clusters, three type II PKS gene clusters, two type III PKS gene clusters, six NRPS gene clusters, and one hybrid PKS/NRPS gene cluster. The type II PKS gene cluster including 2-amino-3-hydroxycyclopent-2-enone synthetic genes was identified to be responsible for hyaluromycin synthesis. We propose the biosynthetic pathway based on bioinformatic analysis.

## Introduction

Hyaluromycin is a hyaluronidase inhibitor isolated from the culture broth of an actinomycete strain MB-PO13^T^ of the genus *Streptomyces* [1]. The structure consists of a γ-rubromycin core possessing a C_5_N unit as an amide substituent of the carboxyl functionality. Rubromycins have inhibitory activities against human telomerase and the reverse transcriptase of human immunodeficiency virus-1 [2]. The core structure possesses a hexacyclic ring system and a 5,6-bisbenzannelated spiroketal structure. The most intriguing part of hyaluromycin is the C_5_N moiety, which is present only in a limited range of secondary metabolites of actinomycetes [3]. As for the rubromycin family biosynthesis, putative biosynthetic genes for griseorhodin A were reported [4], but there is no report on the rubromycins. Hence, the biosynthesis of rubromycin family remains unclear. In this study, we performed whole genome shotgun sequencing of the strain MB-PO13^T^ to elucidate the biosynthetic mechanism of hyaluromycin. We herein present the draft genome sequence of *Streptomyces hyaluromycini* MB-PO13^T^, together with the taxonomical identification of the strain, description of its genome properties and annotation of the gene cluster for hyaluromycin synthesis. The biosynthetic pathway of hyaluromycin is also proposed on the basis of the bioinformatic prediction.

## Organism information

### Classification and features

**Fig. 1:**
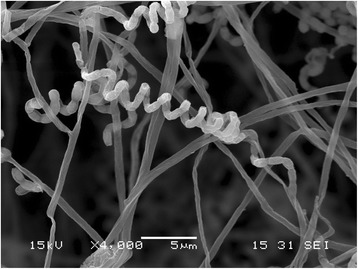
Scanning electron micrograph of *Streptomyces hyaluromycini* MB-PO13^T^ grown on 1/10 ISP 2 agar for 14 days at 28 °C. Bar, 5 μm

**Fig. 2:**
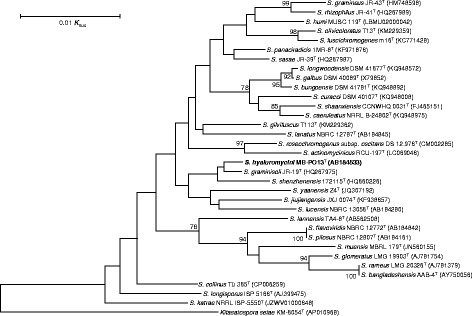
Neighbor-joining phylogenetic tree based on 16S rRNA gene sequences of strain MB-PO13^T^ and its taxonomic neighbors. *Kitasatospora setae* KM-6054^T^ (AB022868) was used as the outgroup. Bootstrap values (>70%) based on 1000 replicates are shown at branch nodes. Bar, 0.01 substitutions per nucleotide position

During the course of screening for hyaluronidase inhibitors from actinomycetes, *Streptomyces hyaluromycini* MB-PO13^T^ was isolated from a tunicate (*Molgula manhattensis*) collected in Tokyo Bay, Japan and found to produce hyaluromycin [1]. Colony appearance was examined after incubation at 28 °C for 14 days on an agar plate of ISP 4. Morphological features were observed under a light microscope (model BX-51; Olympus) and a scanning electron microscope (model JSM-6060; JEOL). The temperature range and optimum temperature for growth were determined by incubating the strain at 5, 10, 15, 20, 28, 37, 42, and 50 °C on ISP 2 agar plates for 14 days. The pH range for growth was determined at 28 °C in ISP 2 broth, of which pH was adjusted to 3 to 12 by 1 N HCl or 1 M Na_2_CO_3_. Tolerance to NaCl was tested on ISP 2 agar plates containing 2, 3, 5, 7, 9, and 12% (*w*/*v*) NaCl at 28 °C. Carbohydrate utilization was determined on ISP 9 supplemented with sterilized carbon sources [5]. The strain grow well on ISP 3, ISP 4 and yeast-starch agars but poor on ISP 2, ISP 5, ISP 6, ISP 7, glucose-asparagine, nutrient, sucrose-nitrate and skim milk agars. Soluble red pigments are produced on ISP 2, ISP 3, ISP 4, ISP 7, glucose-asparagine, nutrient and yeast-starch agars. Cells are aerobic and Gram-stain-positive. The aerial mycelia are branched and yellowish white in color, which become light grey at sporulation and the substrate mycelia are deep red on ISP 4 agar plate. Smooth surface spores (0.5–0.8 × 1.0–1.5 μm) in spiral chains are formed when cultured on nutritionally poor media. A scanning electron micrograph of the strain is shown in Fig. 1. Growth occurs at 10–37 °C (optimum 28 °C), at pH 4.0–9.0 (optimum pH 7.0) and in the presence of less than 2% NaCl (*w*/*v*). The strain utilizes L-arabinose, D-fructose, D-glucose, inositol, D-mannitol, rhamnose and D-xylose as sole carbon source for energy and growth, but not raffinose and sucrose (all at 1%, w/v). These results are summarized in Table 1. The genes encoding 16S rRNA were amplified by PCR using two universal primers, 27F (5′-AGAGTTTGATCMTGGCTCAG-3′) and 1492R (5′-TACGGYTACCTTGTTACGACTT-3′) [6]. GoTaq Green Master Mix (Promega) was used as described by the manufacture for the PCR. The reaction was started with denaturation at 94 °C for 5 min followed by a total 27 cycles that consisted of denaturation at 94 °C for 30 s, annealing at 57 °C for 30 s, and extension at 72 °C for 1.5 min, and extension at 72 °C for 7 min. The PCR product was purified by Wizard SV Gel and PCR Clean-Up System (Promega) and sequenced with a BigDye cycle sequencing ready reaction kit (Appled Biosystems) on an ABI PRISM 310 Genetic analyzer (Applied Biosystems). The sequence was deposited into DDBJ under the accession number AB184533. BLAST search of the sequence by the EzTaxon-e server [7] indicated the highest similarity to that of *Streptomyces graminisoli* JR-19^T^ (HQ267975, 99.79%, 1440/1443). A phylogenetic tree was reconstructed on the basis of the 16S rRNA gene sequence together with taxonomically close *Streptomyces* type strains using CLUSTAL-W program [8] and by the neighbor-joining method [9] using the MEGA 6.0 program [10]. The resultant tree topologies were evaluated by bootstrap analysis [11] based on 1000 replicates. The phylogenetic tree is shown in Fig. 2. On the basis of these findings, strain MB-PO13^T^ was proposed to be classified as a representative of a novel species of the genus *Streptomyces*, with the name *Streptomyces hyaluromycini* sp. nov. [12].

**Table 1:** Classification and general features of *Streptomyces hyaluromycini* MB-PO13^T^

MIGS ID	Property	Term	Evidence code^a^
	Classification	Domain *Bacteria*	TAS [24]
		Phylum *Actinobacteria*	TAS [25]
		Class *Actinobacteria*	TAS [26]
		Order *Actinomycetales*	TAS [26–29]
		Suborder *Streptomycineae*	TAS [26, 29]
		Family *Streptomycetaceae*	TAS [26, 28–31]
		Genus *Streptomyces*	TAS [28, 31–33]
		Species *Streptomyces hyaluromycini*	TAS [12]
		Strain: MB-PO13	TAS [1]
	Gram stain	Gram-positive	TAS [12]
	Cell shape	Branched mycelia	TAS [12]
	Motility	Not reported	
	Sporulation	Sporulating	TAS [12]
	Temperature range	10 °C to 37 °C	TAS [12]
	Optimum temperature	28 °C	TAS [12]
	pH range; Optimum	4 to 9; 7	TAS [12]
	Carbon source	Glucose, inositol, arabinose, fructose, glucose, inositol, mannitol, rhamnose, xylose	TAS [12]
MIGS-6	Habitat	Tunicate (*Molgula manhattensis*)	TAS [1]
MIGS-6.3	Salinity	0% to 2% NaCl	TAS [12]
MIGS-22	Oxygen requirement	Aerobic	TAS [12]
MIGS-15	Biotic relationship	Free-living	TAS [12]
MIGS-14	Pathogenicity	Not reported	
MIGS-4	Geographic location	Tokyo Bay, Minato-ku, Tokyo, Japan	TAS [1]
MIGS-5	Sample collection	August 13, 2007	NAS
MIGS-4.1	Latitude	35° 37′ 33″ N	NAS
MIGS-4.2	Longitude	139° 45′ 5″ E	NAS
MIGS-4.4	Altitude	−1.0 m. above sea level	NAS

^a^ Evidence codes - IDA: Inferred from Direct Assay; TAS: Traceable Author Statement (i.e., a direct report exists in the literature); NAS: Non-traceable Author Statement (i.e., not directly observed for the living, isolated sample, but based on a generally accepted property for the species, or anecdotal evidence). These evidence codes are from the Gene Ontology project [34]

#### Chemotaxonomic data

The isomer of diaminopimelic acid in the whole-cell hydrolysate was analyzed according to the method described by Hasegawa et al. [13]. Isoprenoid quinones and cellular fatty acids were analyzed as described previously [14]. The whole-cell hydrolysate of strain MB-PO13^T^ contained LL-A_2_pm, glucose and mannose. The detected menaquinones were identified as MK-9(H_8_), MK-9(H_6_), MK-9(H_4_) and MK-9(H_10_) (5:37:57:1). The principal polar lipids were diphosphatidylglycerol, phosphatidylethanolamine and phosphatidylinositol. Six unidentified phospholipids were also detected. The major cellular fatty acids (>10%) were *anteiso*-C_15:0_ (24.9%), *iso*-C_16:0_ (23.4%), *iso*-C_14:0_ (15.0%) and C_16:0_ (10.7%). These chemotaxonomic features corresponded to those of the genus *Streptomyces*.

## Genome sequencing information

### Genome project history

In collaboration between Toyama Prefectural University and NBRC, the organism was selected for genome sequencing to elucidate the hyaluromycin biosynthetic pathway. We successfully accomplished the genome project of *Streptomyces hyaluromycini* MB-PO13^T^ as reported in this paper. The draft genome sequences have been deposited in the INSDC database under the accession number BCFL01000001-BCFL01000052. The project information and its association with MIGS version 2.0 compliance are summarized in Table 2 [15].

**Table 2:** Project information

MIGS ID	Property	Term
MIGS 31	Finishing quality	High-Quality Draft
MIGS-28	Libraries used	454 shotgun library, Illumina paired-end library
MIGS 29	Sequencing platforms	454 GS FLX+, Illumina HiSeq1000
MIGS 31.2	Fold coverage	77×
MIGS 30	Assemblers	Newbler v2.6, GenoFinisher
MIGS 32	Gene calling method	Prodigal
	Locus Tag	MB-PO13
	Genbank ID	BCFL01000001-BCFL01000052
	GenBank Date of Release	July 1, 2017
	GOLD ID	Not registered
	BIOPROJECT	PRJDB4283
MIGS 13	Source Material Identifier	NBRC 110483
	Project relevance	Industrial

### Growth conditions and genomic DNA preparation

*Streptomyces hyaluromycini* MB-PO13^T^ was deposited in the NBRC culture collection with the registration number of NBRC 110483^T^. Its monoisolate was grown on polycarbonate membrane filter (Advantec) on 1/2 ISP 2 agar medium (0.2% yeast extract, 0.5% malt extract, 0.2% glucose, 2% agar, pH 7.3) at 28 °C. High quality genomic DNA for sequencing was isolated from the mycelia with an EZ1 DNA Tissue Kit and a Bio Robot EZ1 (Qiagen) according to the protocol for extraction of nucleic acid from Gram-positive bacteria. The size, purity, and double-strand DNA concentration of the genomic DNA were measured by pulsed-field gel electrophoresis, ratio of absorbance values at 260 nm and 280 nm, and Quant-iT PicoGreen dsDNA Assay Kit (Life Technologies), respectively, to assess the quality of genomic DNA.

### Genome sequencing and assembly

Shotgun and paired-end libraries were prepared and subsequently sequenced using 454 pyrosequencing technology and HiSeq1000 (Illumina) paired-end technology, respectively (Table 2). The 77 Mb shotgun sequences and 881 Mb paired-end sequences were assembled using Newbler v2.8 and subsequently finished using GenoFinisher [16] to yield 52 scaffolds larger than 500 bp.

### Genome annotation

Coding sequences were predicted by Prodigal [17] and tRNA-scanSE [18]. The gene functions were annotated using an in-house genome annotation pipeline, and PKS and NRPS-related domains were searched using the SMART and PFAM domain databases. PKS and NRPS gene clusters were determined as reported previously [19]. BLASTP search against the NCBI nr databases were also used for predicting function of proteins encoded in the hyaluromycin biosynthetic gene cluster.

### Genome properties

The total size of the genome of *Streptomyces hyaluromycini* MB-PO13^T^ is 11,525,033 bp and the GC content is 71.0% (Table 3), similar to other genome-sequenced *Streptomyces* members such as *Streptomyces violaceoniger* Tu4133, *Streptomyces* *bingchenggensis* BCW-1 [20] and *Streptomyces rapamycinicus* NRRL 5491^T^. Of the total 10,201 genes, 10,098 are protein-coding genes and 103 are RNA genes. The classification of genes into COGs functional categories is shown in Table 4. As for secondary metabolite pathways by PKSs and NRPSs, *Streptomyces hyaluromycini* MB-PO13^T^ has at least six type I PKS gene clusters, three type II PKS gene clusters, two type III PKS gene clusters, six NRPS gene clusters, and one hybrid PKS/NRPS gene cluster.

**Table 3:** Genome statistics

Attribute	Value	% of Total
Genome size (bp)	11,525,033	100.0
DNA coding (bp)	10,176,135	88.3
DNA G + C (bp)	8,184,694	71.0
DNA scaffolds	52	–
Total genes	10,201	100.0
Protein coding genes	10,098	99.0
RNA genes	103	1.0
Pseudo genes	–	–
Genes in internal clusters	4827	47.3
Genes with function prediction	7049	69.1
Genes assigned to COGs	5317	52.1
Genes with Pfam domains	7836	77.6
Genes with signal peptides	1003	9.9
Genes with transmembrane helices	2326	23.0
CRISPR repeats	2	0

**Table 4:** Number of genes associated with general COG functional categories

Code	Value	%age	Description
J	244	2.4	Translation, ribosomal structure and biogenesis
A	0	0	RNA processing and modification
K	948	9.4	Transcription
L	129	1.3	Replication, recombination and repair
B	1	0	Chromatin structure and dynamics
D	45	0.4	Cell cycle control, cell division, chromosome partitioning
V	205	2.0	Defense mechanisms
T	477	4.7	Signal transduction mechanisms
M	279	2.8	Cell wall/membrane biogenesis
N	25	0.2	Cell motility
U	24	0.2	Intracellular trafficking and secretion
O	176	1.7	Posttranslational modification, protein turnover, chaperones
C	397	3.9	Energy production and conversion
G	563	5.6	Carbohydrate transport and metabolism
E	480	4.8	Amino acid transport and metabolism
F	108	1.1	Nucleotide transport and metabolism
H	332	3.3	Coenzyme transport and metabolism
I	497	4.9	Lipid transport and metabolism
P	281	2.8	Inorganic ion transport and metabolism
Q	380	3.8	Secondary metabolites biosynthesis, transport and catabolism
R	708	7.0	General function prediction only
S	82	0.8	Function unknown
–	4781	47.3	Not in COGs

The total is based on the total number of protein coding genes in the genome

## Insights from the genome sequence

### Hyaluromycin biosynthetic pathway in *Streptomyces hyaluromycini* MB-PO13^T^

**Fig. 3:**
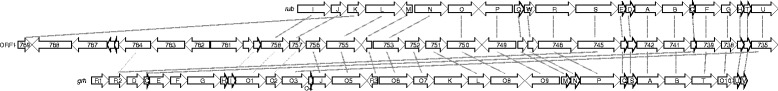
Gene organizations of rubromycin-, hyarulomycin- and griseorhodin-biosynthetic gene clusters. Homologs are linked by gray dotted lines. The *rub*, Orf1- and *grh* are rubromycin-, hyarulomycin- and griseorhodin-biosynthetic gene clusters, respectively. Hyarulomycin-biosynthetic genes are indicated with orf numbers as shown in Table 5

**Fig. 4:**
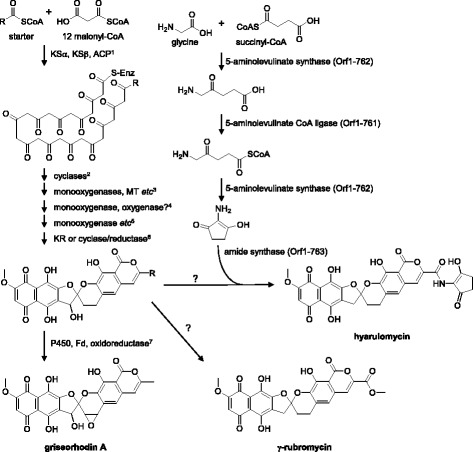
Putative biosynthetic pathways of hyarulomycin, rubromycin and griseorhodin. Each step is catalyzed by enzymes encoded following genes as proposed in griseorhodin biosynthesis [4]. ^1^*grhA*/*rubA*/orf1-742 (KSα), *grhB*/*rubB*/orf1-741 (KSβ) and *grhC*/*rubC*/orf1-740 (ACP); ^2^*grhE*/*rubK*?/orf1-769?, *grhQ*/*rubE*/orf1-744, *grhS*/*rubD*/orf1-743 and *grhT*/*rubF*/orf1-739, ^3^*grhO8*/*rubO*/orf1-750, *grhO9*/*rubP*/orf1-749 (monooxygenases), *grhL*/−/orf1-751 (MT), *grhM*/*rubQ*/orf1-748 (unknown) and *grhP*/*rubR*/orf1-746 (asparagine synthase); ^4^*grhO5*/*rubL*/orf1-755 (monooxygenase) and *grhO1*/*rubI*/orf1-758 (oxygenase)?; ^5^*grhO6*/*rubN*/orf1-753 (monooxygenase) and *grhJ*/−/orf1-756 (acetyltransferase)?; ^6^*grhO10*/*rubG*/orf1-738 (KR) or *grhT*/*rubF*/orf1-739 (cyclase/reductase); ^7^*grhO3*/*rubU*/orf1-735 (cytochrome P450), *grhO4*/−/− (ferredoxin) and *grhO7*/−/orf1-752 (oxidoreductase). Homologs are connected with slashes in order of rubromycin/griseorhodin/hyarulomycin. ACP, acyl carrier protein; CLF, chain length factor; Fd, ferredoxin; KS, ketosynthase; KR, ketoreductase; MT, methyltransferase; −, no homolog in the sequence

Hyarulomycin is a derivative of γ-rubromycin, possessing a C_5_N unit instead of a methoxy group as a side chain. The rubromycin-biosynthetic (*rub*) gene cluster is published in the GenBank (accession no. AF293355.2), but the biosynthetic mechanism has not been reported yet. Among the members of rubromycin family, only the griseorhodin-biosynthetic (*grh*) pathway has been extensively studied: griseorhodin A is synthesized by type II PKSs and modification enzymes [4, 21]. In the genome sequence of *S. hyaluromycini* MB-PO13^T^, three type II PKS gene clusters are present. Among them, the type II PKS gene cluster in scaffold000001 resembles those of rubromycin and griseorhodin as shown in Fig. 3 and Table 5. But, unlike *rub* and *grh* gene clusters, the cluster also encodes amide synthase (Orf1-763), 5-aminolevulinate synthase (Orf1-762) and AMP-dependent synthase (Orf1-761) essential for C_5_N unit synthesis [22]. Thus, we considered it to be the biosynthetic gene cluster for hyarulomycin. According to the proposed biosynthetic mechanisms of griseorhodin [4] and C_5_N [22, 23], we predicted the biosynthetic pathway of hyarulomycin as shown in Fig. 4. The polyketide chain is synthesized by the iterative condensation of an acyl-CoA starter and 12 malonyl-CoA units. This elongation cycle is catalyzed by KSα, KSβ (chain length factor) and acyl carrier protein. Since almost all the homologs of Grh enzymes are present in the putative hyarulomycin-biosynthetic gene cluster (Table 5, Fig. 3), the resulting polyketide chain is likely cyclized and modified to the polycyclic intermediate bearing a spiroketal moiety in the similar fashion to griseorhodin biosynthesis. Unlike griseorhodin A, the epoxide functionality is not present in the spiroketal moiety of rubromycin and hyaluromycin. This can be explained by the absence of  homolog of *grhO4* encoding ferredoxin responsible for epoxide formation of griseorhodin A in rubromycin- and hyarulomycin-biosynthetic gene clusters. It was unable to predict a gene responsible for the removal of the hydroxyl group at the spiroketal only by this bioinformatic analysis. 5-Aminolevulinate synthase (Orf1-762), 5-aminolevulinate CoA ligase (Orf1-761) and amide synthase (Orf1-763) are involved in the formation of C_5_N unit and its coupling with the aromatic core.

**Table 5:** Putative hyaluromycin biosynthetic gene cluster and the neighboring genes

Orf1-	Size (aa)	Proposed function	Closest homolog		Homolog (I/S, %) in	
			Description, *Origin*, Accession number	I/S^b^ (%)	*grh* cluster	*rub* cluster
769	230	cyclase	hypothetical protein, *Streptomyces fulvoviolaceus*, WP_052425082	54/63	–	RubK (53/63)
768^a^	656	ABC transporter ATP-binding protein	multidrug ABC transporter ATP-binding protein, *Actinopolymorpha alba*, WP_020576731	70/83	–	–
767^a^	577	multidrug ABC transporter ATPase	multidrug ABC transporter ATPase, *Streptomyces varsoviensis*, WP_030881385	69/81	–	–
766^a^	117	MarR family transcriptional regulator	MarR family transcriptional regulator, *Actinomadura macra*, WP_067468911	45/63	–	–
765^a^	72	unknown	hypothetical protein, *Streptomyces aurantiacus*, WP_055507532.	56/60	–	–
764^a^	498	transcriptional regulator	hypothetical protein, *Streptomyces* sp. NRRL WC-3742, WP_051836320	55/63	GrhR2 (34/48)	
763^a^	533	amide synthetase	hypothetical protein, partial, *Streptomyces* sp. NRRL WC-3742, WP_078910860	60/70	–	–
762^a^	405	5-aminolevulinate synthase	AsuD2, *Streptomyces nodosus* subsp. *asukaensis*, ADI58646	77/85	–	–
761	515	5-aminolevulinate CoA ligase	AMP-dependent synthetase, *Streptomyces uncialis*, OKH94380	77/83	–	–
760	183	unknown	hypothetical protein, *Streptomyces prunicolor*, WP_019061819	50/60	–	–
759	122	unknown	hypothetical protein, *Streptomyces fulvoviolaceus*, WP_030615859	72/82	GrhI (61/73)	–
758	477	oxygenase	hypothetical protein, *Streptomyces yerevanensis*, WP_033324694	72/82	GrhO1 (72/80)	RubI (71/80)
757	257	3-oxoacyl-ACP reductase	SDR family oxidoreductase, *Streptomyces fulvoviolaceus*, WP_030615854	83/92	GrhO2 (73/81)	RubJ (83/91)
756	325	acetyltransferase	GrhJ, *Streptomyces* sp. CN48+, AIE76926	68/74	GrhJ (67/73)	–
755	540	monooxygenase	hypothetical protein, *Streptomyces prunicolor*, WP_026151147	73/80	GrhO5 (69/75)	RubL (73/80)
754^a^	161	transcriptional regulator	putative transcriptional repressor GrhR3, *Streptomyces* sp. CN48+, AIE76928	76/88	GrhR3 (76/88)	RubM (74/83)
753	501	monooxygenase	RubN, *Streptomyces collinus*, AAM97364	80/86	GrhO6 (73/80)	RubN (80/86)
752	325	oxidoreductase	hypothetical protein, *Streptomyces* sp. TSRI0261, WP_073806081	86/93	GrhO7 (78/89)	–
751	343	methyltransferase	hypothetical protein, *Streptomyces fulvoviolaceus*, WP_030615823	81/86	GrhL (77/83)	–
750	535	monooxygenase	hypothetical protein, *Streptomyces prunicolor*, WP_019061807	74/82	GrhO8 (70/79)	RubO (63/72)^p^
749^a^	534	oxidoreductase	hypothetical protein, *Streptomyces* sp. TP-A0875, WP_053912978	74/80	GrhO9 (71/79)	RubP (74/80)
748	161	unknown	hypothetical protein, *Streptomyces prunicolor*, WP_019061805	81/85	GrhM (80/86)	RubQ (80/85)
747	174	unknown	hypothetical protein, *Streptomyces fulvoviolaceus*, WP_030615810	67/74	GrhN (56/64)	RubW (64/74)
746	623	asparagine synthase	RubR, *Streptomyces collinus*, AAM97368	80/86	GrhP (74/81)	RubR (80/86)
745	669	transcriptional regulator	RubS, *Streptomyces collinus*, AAM97369	63/75	GrhR2 (43/56)	RubS (63/75)
744	123	cyclase	putative cyclaseI, *Streptomyces collinus*, AAG03065	83/88	GrhQ (75/88)	RubE (83/88)
743	143	cyclase	cupin, *Streptomyces* sp. TSRI0261, OKJ01252	83/90	GrhS (66/77)	RubD (79/85)
742	424	ketosynthase α subunit	type II polyketide synthase 4, *Streptomyces* sp., APD71740	89/95	GrhA (85/91)	RubA (89/93)
741	420	ketosynthase β subunit	type II polyketide synthase 5, *Streptomyces* sp., APD71741	82/88	GrhB (76/83)	RubB (79/85)
740	87	acyl carrier protein	acyl carrier protein, *Streptomyces collinus*, AAG03069	68/79	GrhC (34/61)	RubC (68/79)
739	398	cyclase/reductase	hypothetical protein, *Streptomyces prunicolor*, WP_019061796	79/87	GrhT (67/78)	RubF (78/85)
738	249	ketoreductase	SDR family oxidoreductase, *Streptomyces prunicolor*, WP_019061795	86/94	GrhO10 (79/89)	RubG (86/93)
737	108	monooxygenase	hypthetical protein, *Streptomyces collinus*, AAG03072	88/93	GrhU (75/84)	RubH (88/93)
736	113	unknown	hypothetical protein, *Streptomyces fulvoviolaceus*, WP_078655944	73/80	GrhV (67/76)	RubT (70/81)
735	417	cytochrome P450	cytochrome P450, *Streptomyces fulvoviolaceus*, WP_030615776	80/86	GrhO3 (37/53)	RubU (80/86)
734	301	unknown	DUF1963 domain-containing protein, *Streptacidiphilus carbonis*, WP_042397320	78/85	–	–
733	155	cupin	cupin, *Streptomyces prunicolor*, WP_019056246	93/97	–	–
732	322	esterase	alpha/beta hydrolase, *Actinobacteria* bacterium OK074, KPI24488	83/88	–	–
731	313	transcriptional regulator	transcriptional regulator, *Streptomyces hokutonensis*, WP_043260174	79/85	–	–
730^a^	491	unknown	dolichyl-phosphate-mannose-protein mannosyltransferase, *Micromonospora auratinigra*, SBT53146	57/67	–	–
729	42	unknown	–	–	–	–
728^a^	333	transcriptional regulator	LacI family transcriptional regulator, ‘*Streptomyces humi’*, WP_046734674	93/96	–	–

^a^encoded in complementary strand, ^b^I/S, identity/similarity. Orf1-763 also shows 48% sequence identity/61% sequence similarity to AsuD1 of *Streptomyces nodosus* subsp. *asukaensis* (ADI58645); Orf1-761 shows 73% sequence identity/81% sequence similarity to AsuD3 of *S. nodosus* subsp. *asukaensis* (ADI58647)

## Conclusions

The 11.5 Mb draft genome of *Streptomyces hyaluromycini* MB-PO13^T^, a producer of hyaluromycin, isolated from tunicate (*Molgula manhattensis*) has been deposited at GenBank/ENA/DDBJ under the accession number BCFL00000000. We successfully identified the gene cluster for hyaluromycin synthesis and proposed the plausible biosynthetic pathway. These findings provide useful information for genetic engineering to synthesize more potential hyaluronidase inhibitors and discovering new bioactive aromatic polyketides possessing the C_5_N unit.

## Abbreviations

A_2_pm: Diaminopimelic acid
ABC: ATP-binding cassette
ACP: Acyl carrier protein
C_5_N: 2-amino-3-hydroxycyclopent-2-enone
CLF: Chain length factor
CoA: Coenzyme A
DDBJ: DNA Data Bank of Japan
Fd: Ferredoxin
ISP: International *Streptomyces* project
KR: Ketoreductase
KS: Ketosynthase
MK: Menaquinone
MT: Methyltransferase
NBRC: Biological Resource Center, National Institute of Technology and Evaluation
NRPS: Nonribosomal peptide synthetase
PKS: Polyketide synthase
